# Quarterly repeat cycles of onabotulinumtoxinA in chronic migraine patients: the benefits of the prolonged treatment on the continuous responders and quality-of-life conversion rate in a real-life setting

**DOI:** 10.1007/s10072-017-3054-y

**Published:** 2017-07-19

**Authors:** Antonio Santoro, Andrea Fontana, Anna M. Miscio, Michele M. Zarrelli, Massimiliano Copetti, Maurizio A. Leone

**Affiliations:** 1 Unit of Neurology, IRCCS Casa Sollievo della Sofferenza, San Giovanni Rotondo, FG Italy; 2 Unit of Biostatistics, IRCCS Casa Sollievo della Sofferenza, San Giovanni Rotondo, FG Italy

**Keywords:** Chronic migraine, Medication overuse headache, Migraine abuse, Preventative therapy, OnabotulinumtoxinA

## Abstract

OnabotulinumtoxinA was approved for treatment of chronic migraine (CM) after publication of Phase 3 Research Evaluating Migraine Prophylaxis Therapy (PREEMPT) trials. However, the PREEMPT trials lasted only up to 1 year. The main aim of our retrospective study was to evaluate whether a prolonged treatment of onabotulinumtoxinA (18 months, six quarterly cycles) will sustain or further improve the efficacy results and the quality of life achieved at 6 and 12 months. Patients were adults with CM with or without overuse of drugs, with at least six regularly repeat onabotulinumtoxinA treatments, administered according to the PREEMPT protocol. The outcomes were investigated after 6, 12, and 18 months of treatment with respect to baseline and with respect to each previous study time point. Headache days and hours, and dosage of headache medication taken with latency period, were collected from the patients daily. Quality of life was evaluated by means of the Migraine Disability Assessment (MIDAS) questionnaire. At each study time point, the proportion of responder patients with respect to baseline was evaluated. For all measures, the baseline data were referred to the previous month before starting. Forty-seven patients were evaluated. Our data show a decrease in the monthly headache days and hours, at each study evaluation, with respect to the previous one. They showed that beyond the first year, a statistically significant difference in the monthly days of headache compared at 18 vs. 12 months is observed. A significantly higher proportion of patients (with a response greater than 75% decrease from baseline in the frequency of headache days and hours) was observed at month 18 compared to month 12. The proportion of patients in MIDAS grade I increased over time, and a statistically significant improvement in MIDAS I score was obtained from month 12 to month 18. A positive modification in the consumption of analgesics over time was observed (*p* for trend <0.001). The mean acute drug latency strongly decreased over time. Our study confirmed that onabotulinumtoxinA is an effective treatment to reduce headache-related disability and improve patients’ quality of life, highlighting that upon repeated administration, the therapy efficacy increases significantly and a progressive trend of “first-time response” is observed for the entire period under consideration.

## Background

Chronic migraine (CM) is a common and debilitating neurological disorder affecting up to 2.4% of the general population and with an incidence estimated to be 2.5% per year [1].

According to the International Classification of Headache Disorders (ICHD-III), CM is defined as headache on 15 or more days per month for more than 3 months (at least 8 days should meet criteria for migraine without aura or respond to migraine-specific treatment) [1, 2].

CM is associated with significant disability and reduced health-related quality of life [3, 4]. Moreover, persons with CM have a greater economic burden than patients with episodic migraine (EM) [5–7].

Risk factors for the chronification of migraine have been identified, such as female gender, older age, Caucasian ethnicity, low socioeconomic status, comorbidity with other chronic diseases like obesity or psychiatric disorders, high-frequency EM, and overuse of symptomatic medication [1, 8].

Many patients with CM take a high amount of abortive medications. It is estimated that around 50–80% of patients with CM show an analgesic overuse that may induce medication overuse headache (MOH) [9]. There is no general agreement as to whether MOH is a consequence or a cause of CM [10]. As for CM, psychopathological comorbidities are often present among patients with MOH, and quality of life is highly related to CM as well as to relapse into MOH [11, 12].

Currently, onabotulinumtoxinA is the unique drug specifically indicated for prophylaxis of headache in adult patients with CM [13]. It is the only therapy with an approved indication [14]. Recently, the European Headache Federation recognized the value of onabotulinumtoxinA in CM prevention and specified that before labelling a patient as affected by refractory CM, a proper treatment with this drug needs to be completed [15].

The onabotulinumtoxinA indication is based on the results of two large-scale, placebo-controlled, multicenter trials (PREEMPT (Phase 3 Research Evaluating Migraine Prophylaxis Therapy)).

The Pooled PREEMPT had a 28-day baseline screening phase and a 24-week, 2-cycle, double-blind placebo-controlled, parallel-group phase with two injection cycles, followed by a 32-week, open-label phase with three injection cycles and demonstrated the efficacy, safety, and tolerability of repeated treatment with 155–195 U of onabotulinumtoxinA, every 12 weeks over 56 weeks (up to five treatment cycles), as a prophylactic treatment for CM in adults [16–19].

Nowadays, few studies have been published based on onabotulinumtoxinA real-life efficacy and few data are available on its efficacy beyond the fifth cycle of treatment administered over a period longer than 12 months [20–23].

The main aim of our retrospective study was to evaluate whether in a real-life clinical setting, a prolonged treatment of onabotulinumtoxinA (18 months, six quarterly cycles) will sustain or further improve the efficacy results and the quality of life achieved at 6 and 12 months.

## Methods

### Study cohort

All patients included in our retrospective study were adults, male and female, with CM defined by the International Classification of Headache Disorders (ICHD-3 beta 2013) [24], with (the majority) or without overuse of drugs, with at least six regularly repeat onabotulinumtoxinA treatments.

Our patient cohort had received and failed other preventive therapies due to lack of efficacy or intolerable side effects and was able to fill a specific “migraine diary,” without lack of information.

Exclusion criteria were pregnancy or breastfeeding, symptoms of psychiatric disease, and history of botulinum toxin use for other clinical purposes.

To evaluate a cohort reflecting a real-life clinical setting as closely as possible, patients receiving any preventive or symptomatic therapy of migraine were not excluded from the analysis.

The data were collected from IRCCS Casa Sollievo della Sofferenza—San Giovanni Rotondo—S.C. di Neurologia (from April 2013 to August 2016).

The work carried out complies with the Declaration of Helsinki and was approved by Sezione del CE IRCCS Istituto Tumori Giovanni Paolo II di Bari presso la Fondazione Casa Sollievo della Sofferenza di San Giovanni Rotondo ICF: V1.0_07 APR 2015.

Each patient signed a free informed consent for the analysis and publication of the data.

### Treatment

OnabotulinumtoxinA was injected in a day hospital setting, every 3 months (±10 days) as per the PREEMPT protocol (i.e., 155–195 U onabotulinumtoxinA in 31–39 sites).

### Clinical assessment

All patients were trained to complete a specific headache diary, and they were asked to fill it out at baseline and continuously after receiving onabotulinumtoxinA. Headache days, cumulative hours of headache in headache days, and dosage of headache medication intake, with latency time (time from symptomatic drug administration to the analgesic effect) were daily collected from the patients in the diary and were evaluated by the investigator at each quarterly visit.

The quality of life was evaluated by means of the Migraine Disability Assessment (MIDAS) questionnaire [25] administered at baseline and at each quarterly visit (3 months after each treatment session).

At each visit, the proportion of responder patients with respect to baseline was evaluated.

For all measures, the baseline data were referred to the previous month before starting onabotulinumtoxinA.

### Outcome assessment

The following outcomes were investigated after 6 (T6—i.e., after II cycles), 12 (T12—i.e., after IV cycles) and 18 months (T18—i.e., after VI cycles) of treatment with respect to baseline visit (T0) and with respect to each previous time point (T12 vs. T6; T18 vs. T12):

1. Changes in monthly days and hours of headache
2. Consumption and latency time (in hours) of analgesics
3. MIDAS grade distribution

Moreover, patient treatment responsiveness in terms of the relative reduction (i.e., percentage of responsiveness) for the number of days and hours of headache at T6, T12, and T18 with respect to baseline (T0) and with respect to each study time was investigated.

Such percentage was calculated by subtracting the value of the outcome at T0 with the value of the outcome at follow-up and dividing such difference by the value of the outcome at T0. According to such percentage, patients were therefore classified as follows:

- Non-responder <30% reduction
- Partial responder ≥30 and <50% reduction
- Responder ≥50 and ≤75% reduction
- High responder >75% reduction

### Statistical analysis

Patient characteristics were reported as mean ± standard deviation (SD), median along with lower–upper quartiles (q1–q3) and range (min-max), or frequencies and percentages, for continuous and categorical variables, respectively.

Changes in monthly number of days and hours of headache, MIDAS distribution consumption of analgesics, and drug latency over follow-up time (i.e., at T0, T6, T12, and T18) were assessed using the hierarchical generalized linear model (HGLM) for longitudinal data, for each outcome at issue. Within this framework, the Poisson distribution was assumed to model continuous outcomes concerned with counts (i.e., monthly number of days and hours of headache, drug latency in hours) whereas the logistic distribution was assumed to model binary outcomes (i.e., consumption of analgesics). Changes over time in the distribution of multinomial variables (i.e., MIDAS and responders’ groups) were assessed performing HGLMs with logistic distribution, using indicator variables (i.e., dummy variables) for each category of the variable at issue. The first-order autoregressive covariance structure was used to account for the correlation between repeated measurements over time. Estimated means (or percentages for categorical variables) were carried out from HGLMs and were reported along with their 95% confidence interval (95% CI), including follow-up time as categorical covariate. For each HGLM, a test for overall difference over time was assessed by looking at the significance of the type III test, whereas pairwise comparisons were assessed as statistical contrasts and were adjusted following Benjamini-Hochberg step-down procedure. The presence of a linear trend for the estimated means (or proportions) over time was assessed by looking at the significance of the regression coefficient of the follow-up time variable, included into the model as continuous covariate into HGLMs (*p* for trend). For each continuous outcome at issue, longitudinal plots of the estimated means over time were further reported whereas for each categorical outcome at issue, histograms of the estimated percentages were reported instead. All plots were represented along with error bars which represented 95% CI.

Two-sided *p* values <0.05 were considered for statistical significance. All analyses were performed using SAS Software, Release 9.4 (SAS Institute, Cary, NC, USA) and R (package: ggplot2).

## Results

From April 2013 to August 2016, a total of 207 patients with CM received at least one administration cycle of onabotulinumtoxinA according to the PREEMPT protocol (treatments quarterly, 155–195 U onabotulinumtoxinA in 31–39 sites). Twenty-eight patients discontinued the treatment due to improvement (≤4 headache days and responder to symptomatic treatment for 3 months) before the sixth cycle, and 67 patients were lost to follow-up. Sixty-five patients were ongoing (i.e., before VI onabotulinumtoxinA cycle) when the database was locked for this analysis (August 31, 2016). Finally, 47 patients received six treatments quarterly during 18 months. So, our cohort consisted of 47 patients (37 females, 78.7%) with a mean age of 48.2 ± 13.6 years (range 41–59). Patients had a diagnosis of CM for a mean of 9.4 ± 6.8 years (range 1–30).

Demographic and treatment baseline patient characteristics are reported in Table 1.

**Table 1:** Demographic details of study population at baseline (47 patients)

Age (years)	Mean ± SD Median (q1–q3) Range	48.2 ± 13.6 47 (41–59) 18–72
Sex—females	*n* (%)	37 (78.7)
Years of chronic headache	Mean ± SD Median (q1–q3) Range	9.4 ± 6.8 9 (4–12) 1–30
Patients assuming NSAIDs	*n* (%)	27 (57.5)
Patients assuming triptans	*n* (%)	23 (48.9)
Patients assuming other drugs	*n* (%)	18 (38.3)

### Change of headache days and hours/month

Results from comparisons of monthly mean of headache days at each time point, as well as from comparisons of monthly mean of headache hours, are reported in Table 2.

**Table 2:** Monthly headache days and hours at baseline (T0) and after 6 (T6), 12 (T12), and 18 months (T18)

		T0	T6	T12	T18
Monthly days with headache	Mean ± SD	25.9 ± 5.3	11.5 ± 8.8	9.6 ± 6.8	6.3 ± 5.7
	Median (q1–q3)	30 (20–30)	9 (4–18)	8 (5–14)	5 (2–8)
	Range 95% CI n	15–30 23.0–29.1 47	0–30 9.6–13.8 47	0–30 8.0–11.7 47	0–30 5.0–8.0 47
Monthly hours with headache	Mean ± SD	547.7 ± 183.4	173.4 ± 195.3	90.4 ± 93.9	53.2 ± 79.2
	Median (q1–q3)	600 (400–700)	100 (30–250)	60 (20–120)	25 (12–50)
	Range 95% CI n	112–720 478.4–627.0 47	0–700 136.3–220.5 47	0–400 64.8–126.1 47	0–350 34.5–82.2 47
*p* values from HGLM and pairwise comparisons					
Outcome	*p* for overall difference	T6 vs. T0	T12 vs. T6	T18 vs. T12	*p* for trend
Monthly days with headache	<0.001	<0.001	0.072	0.001	<0.001
Monthly hours with headache	<0.001	<0.001	<0.001	0.013	<0.001

The mean of monthly headache days significantly decreased from 25.9 ± 5.3 at baseline to 11.5 ± 8.8 after II onabotulinumtoxinA cycle (T6), to 9.6 ± 6.8 after IV onabotulinumtoxinA cycle (T12), down to reaching 6.3 ± 5.7 after VI onabotulinumtoxinA cycle (T18) (*p* for trend <0.001). Moreover, a statistically significant decrease of monthly headache days was observed after 6 months with respect to baseline (*p* < 0.001) and after 18 months with respect to 12 months (*p* = 0.001). Also, the mean of monthly headache hours significantly decreased over time (T0, 547.7 ± 183.3; T6, 173.4 ± 195.3; T12, 90.4 ± 93.9; T18, 53.2 ± 79.2) (*p* for trend <0.001). In this case, statistically significant pairwise comparisons were found contrasting all time points: T6 vs. T0 (*p* < 0.001), T12 vs. T6 (*p* < 0.001), and T18 vs. T12 (*p* = 0.013).

**Fig. 1:**
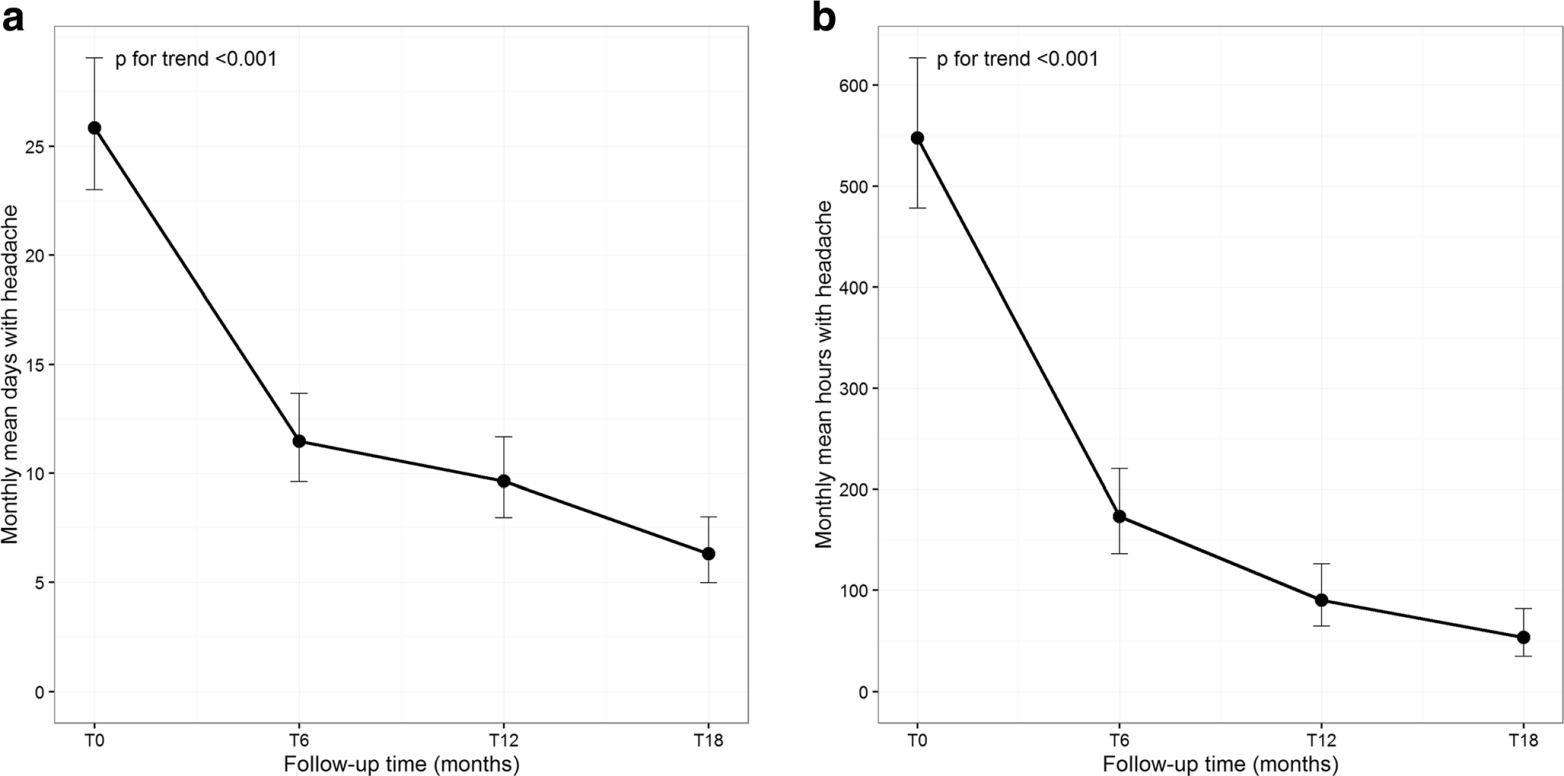
Plots of monthly headache days (**a**) and hours (**b**) means at baseline (T0) and after 6, 12, and 18 months (T6, T12, and T18) of treatment for onabotulinumtoxinA (six quarterly cycles). *Error bars* represent the 95% confidence interval around the means

Plots of monthly headache days and hours means over follow-up time are reported in Fig. 1a, b, respectively.

### Consumption of analgesics and change in mean latency time after acute drug intake

The study results showed also a positive modification in the consumption of analgesics over time (Table 3), pointing out a great decrease of painkiller dosages for each drug class (*p* for trend <0.001).

**Table 3:** Distribution of symptomatic drug (i.e., analgesics) dosage units among users

		T0	T6	T12	T18
NSAID use (total number of dosage units)	Mean ± SD Median (q1–q3) Range 95% CI Users	39.7 ± 31.3 30 (20–55) 3–120 30.6–49.5 27	11.8 ± 15.9 4 (3–15) 2–70 7.8–18.7 25	8.2 ± 9.0 4.5 (2.5–11.5) 1–40 4.7–13.2 28	5.4 ± 6.6 3 (2–6) 1–30 2.8–10.0 28
Triptan use (total number of dosage units)	Mean ± SD Median (q1–q3) Range 95% CI Users	30.4 ± 26.4 24 (15–30) 7–120 21.3–41.7 23	21.9 + 30.6 9 (5–22) 2–120 12.0–29.2 17	8.6 ± 9.5 5.5 (2.5–10) 1–35 4.4–16.1 20	7.6 ± 8.1 5 (3–9) 1–30 3.0–15.0 15
Other drugs use (total number of dosage units)	Mean ± SD Median (q1–q3) Range 95% CI Users	40.1 ± 37.0 30 (9–50) 3–120 28.2–54.0 18	11.9 ± 16.2 6 (3–12) 1–70 7.2–21.4 19	6.6 ± 6.7 4.5 (2.10) 1–30 4.2–15.5 22	5.1 ± 5.8 3 (2–6) 1–22 1.7–13.3 13
*p* values from HGLM and pairwise comparisons					
Outcome	*p* for overall difference	T6 vs. T0	T12 vs. T6	T18 vs. T12	*p* for trend
NSAID use (total number of dosage units)	<0.001	<0.001	0.195	0.205	<0.001
Triptan use (total number of dosage units)	<0.001	0.015	0.005	0.483	<0.001
Other drugs use (total number of dosage units)	<0.001	<0.001	0.241	0.241	<0.001

**Fig. 2:**
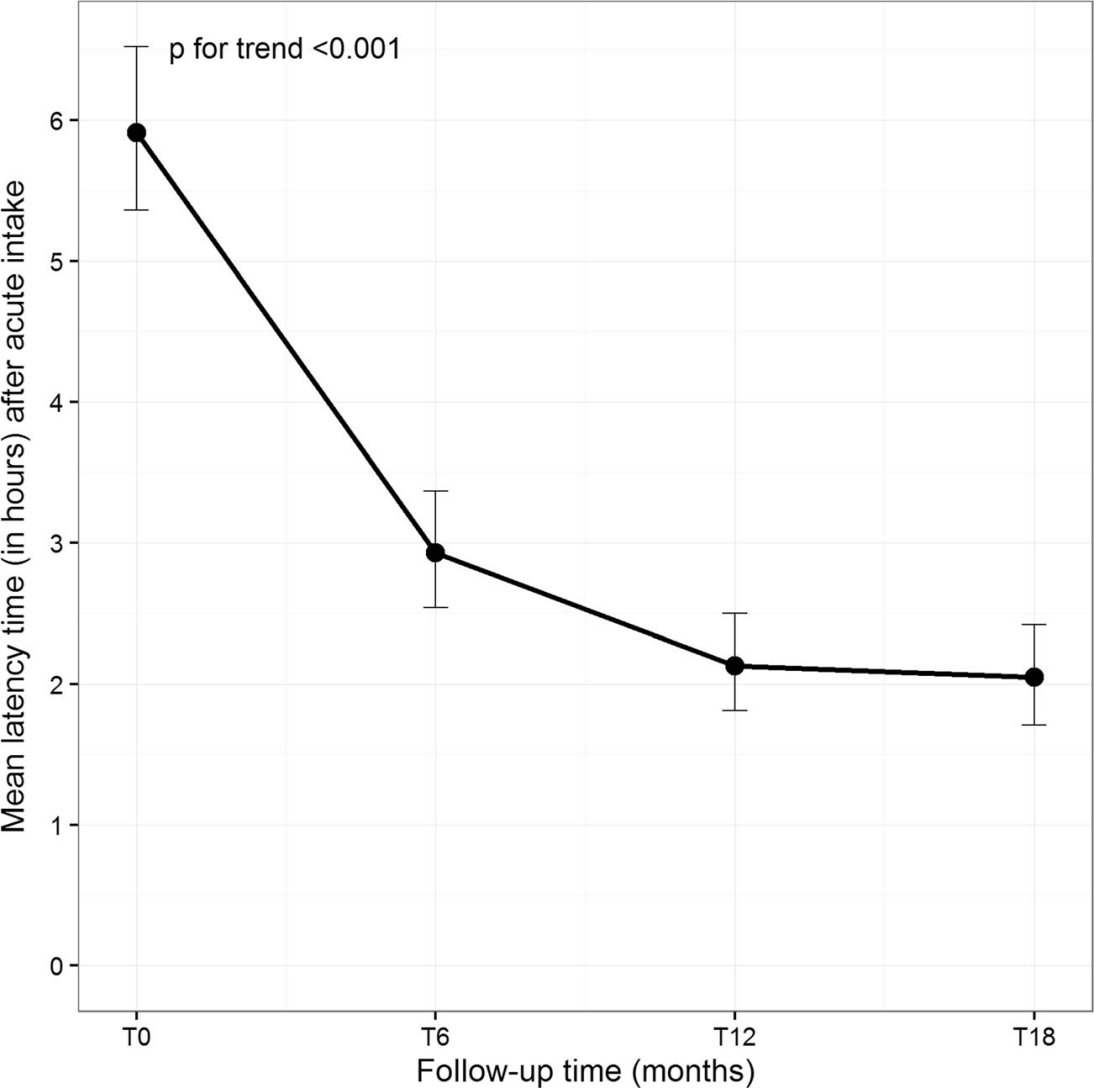
Trend of latency time (h) means after symptomatic drug administration at baseline (T0) and after 6, 12, and 18 months (T6, T12, and T18) of treatment for onabotulinumtoxinA. *Error bars* represent 95% confidence intervals around the means

This was also corroborated with an important and significant decrease in monthly mean time before the effects of any symptomatic medication. The mean acute drug latency strongly decreased at T6 vs. T0 (*p* < 0.001) and at T12 vs. T6 (*p* = 0.001), showing a further slight improvement at T18 (Table 4 and Fig. 2).

**Table 4:** Latency after intake of any symptomatic therapy, among patients who were treated with analgesics (i.e., users)

		T0	T6	T12	T18
Latency time (h)	Mean ± SD	5.9 ± 0.4	2.9 ± 1.5	2.1 ± 1.4	2.0 ± 1.5
	Median (q1–q3)	6 (6–6)	3 (2–4)	2 (1–3)	1.5 (1–2)
	Range 95% CI Users	4–6 5.4–6.5 47	1–6 2.5–3.4 46	1–6 1.8–2.5 47	1–6 1.7–2.4 44
*p* values from HGLM and pairwise comparisons					
	*p* for overall difference	T6 vs. T0	T12 vs. T6	T18 vs. T12	*p* for trend
Latency time (h)	<0.001	<0.001	0.001	0.654	<0.001

### Distribution of patient response to treatment

**Fig. 3:**
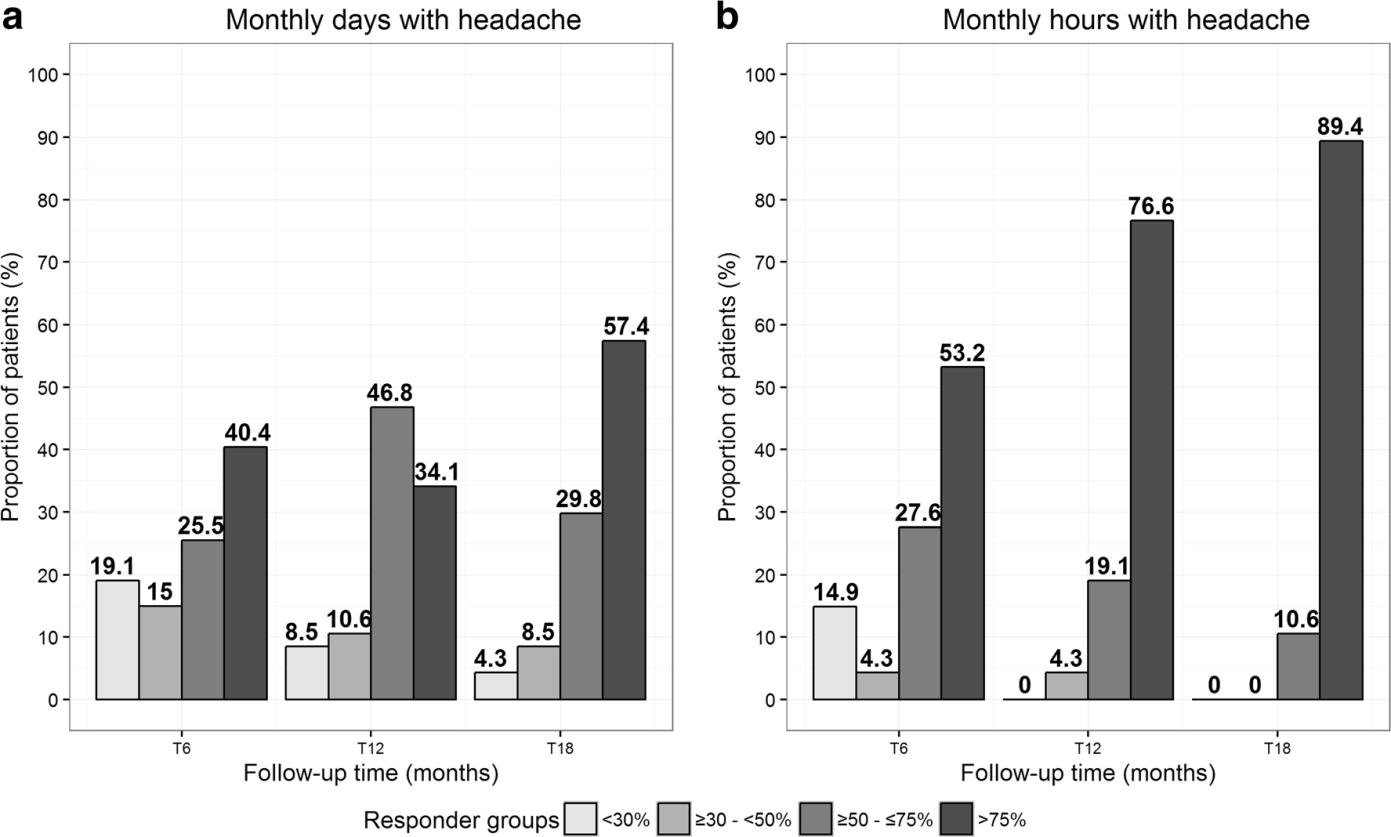
Frequency distribution of patient response to treatment, in terms of number of monthly headache days (**a**) and hours (**b**) with headache at T6, T12, and T18 with respect to baseline visit

Distribution of responder groups (in terms of improvement in monthly days and cumulative hours of headache) at each time point is summarized in Table 5 and is graphically represented in Fig. 3. We observed a strong increase in the proportion of responder patients already after 6 months on either days or hours of headache. As for the monthly days, when classifying responder patients into the three defined categories, we found a significant increase in the proportion of high responders from T12 (34.1%) to T18 (57.4%) (*p* = 0.009). A different scenario was observed when referring to the monthly hours of headache: the proportion of high responders for the hours of headache significantly increased already at T12 vs. T6 (from 53.2% at T6 to 76.6% at T12; *p* = 0.009), and a further enhancement was observed at 18 months (89.4%).

**Table 5:** Distribution of patient response to treatment, in terms of reduction in number of days and hours with headache at each study time (i.e., T6, T12, and T18) with respect to baseline visit (47 patients)

		Follow-up time			*p* values from HGLM and pairwise comparisons				
Outcome	Responder groups	T6 *N* (%)	T12 *N* (%)	T18 *N* (%)	Test for overall difference	T12–T6	T18–T6	T18–T12	Test for trend
Monthly days of headache	Non-responders: reduction <30%	9 (19.1)	4 (8.5)	2 (4.3)	0.078	0.195	0.114	0.344	0.025
	Partial responders: reduction ≥30–<50%	7 (15.0)	5 (10.6)	4 (8.5)	0.570	0.655	0.655	0.655	0.294
	Responders: reduction ≥50–≤75%	12 (25.5)	22 (46.8)	14 (29.8)	0.024	0.047	0.638	0.107	0.667
	High responders: reduction >75%	19 (40.4)	16 (34.1)	27 (57.4)	0.012	0.389	0.140	0.009	0.069
Monthly hours of headache	Non-responders reduction <30%	7 (14.9)	0 (0.0)	0 (0.0)	<0.001	0.006	0.006	1.000	0.074
	Partial-responders: Reduction ≥30–<50%	2 (4.3)	2 (4.3)	0 (0.0)	1.000	1.000	1.000	1.000	0.215
	Responders: Reduction ≥50–≤75%	13 (27.6)	9 (19.1)	5 (10.6)	0.122	0.266	0.121	0.266	0.038
	High responders: reduction >75%	25 (53.2)	36 (76.6)	42 (89.4)	<0.001	0.009	<0.001	0.051	<0.001

### MIDAS grade distribution

**Fig. 4:**
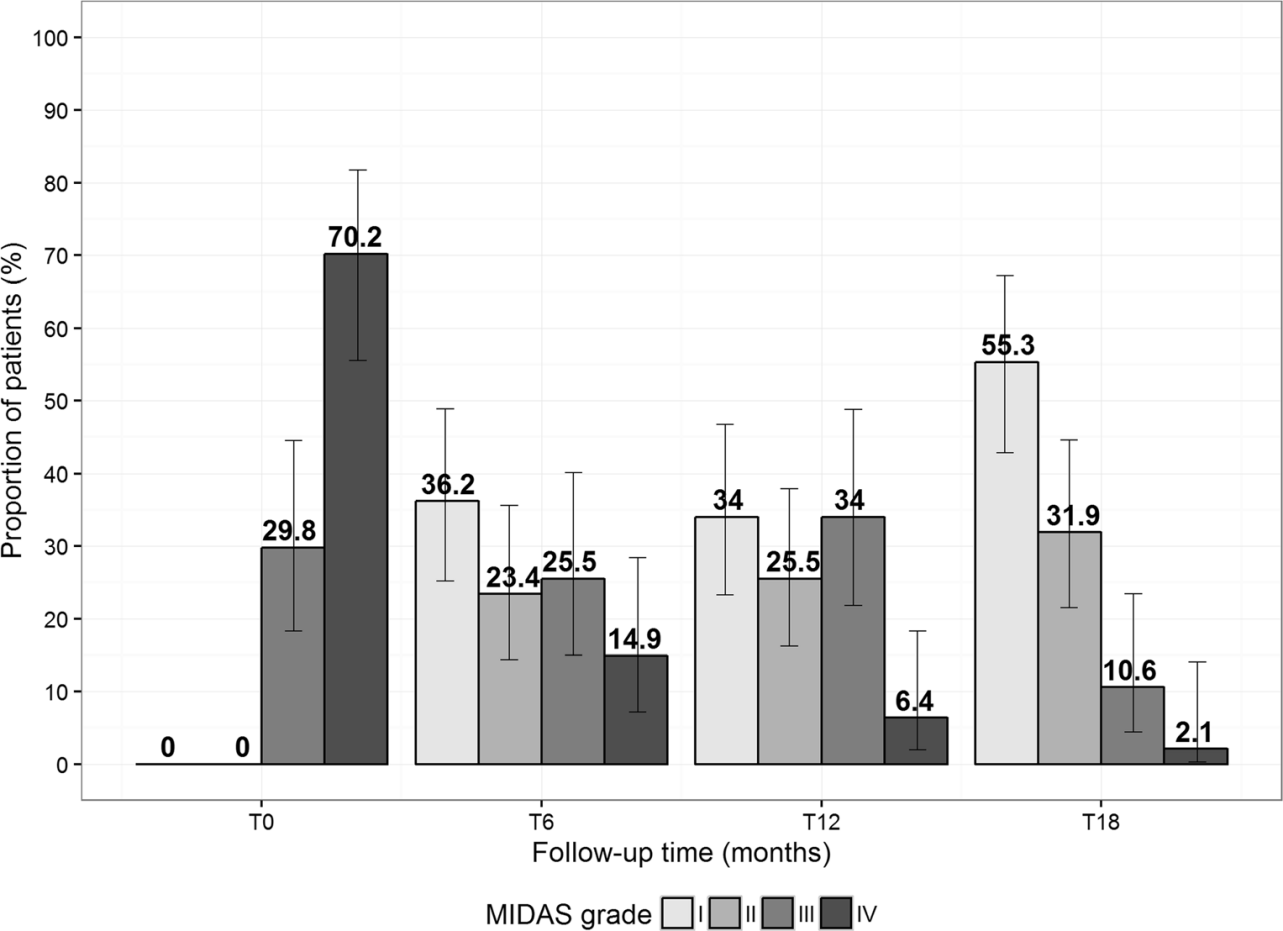
Frequency distribution for MIDAS grade at baseline (T0) and after 6, 12, and 18 months (i.e., T6, T12, and T18). *Error bars* represent 95% confidence interval around proportions

MIDAS grade distribution is represented at each time point in Table 6 and plotted in Fig. 4. The results suggest a significant progressive improvement from baseline to 6–12–18 months, pointing out a high and significant decrease of patients with grade IV already at 6 months compared with baseline (*p* < 0.001), and a constant clinical improvement was confirmed in the subsequent visits (*p* for trend <0.001). At 18 months, a statistically significantly higher proportion of patients with MIDAS grade I compared to those at 12 months (T12, 34.0%; T18, 55.3%; *p* = 0.022) was observed. Conversely, a statistically significantly lower proportion of patients with MIDAS grade III resulted (T12, 34.0%; T18, 10.6%; *p* = 0.017).

**Table 6:** Patient distribution as per MIDAS grades at each study time (47 patients)

		T0	T6		T12	T18
MIDAS grade I	*n* (%) 95% CI	0 (0.0) 0–100	17 (36.2) 25.2–48.9		16 (34.0) 23.3–46.7	26 (55.3) 42.8–67.2
MIDAS grade II	*n* (%) 95% CI	0 (0.0) 0–100	11 (23.4) 14.4–35.6		12 (25.5) 16.2–37.9	15 (31.9) 21.5–44.6
MIDAS grade III	*n* (%) 95% CI	14 (29.8) 18.3–44.5	12 (25.5) 15.0–40.1		16 (34.0) 21.8–48.8	5 (10.6) 4.4–23.4
MIDAS grade IV	*n* (%) 95% CI	33 (70.2) 55.5–81.7	7 (14.9) 7.2–28.4		3 (6.4) 2.0–18.3	1 (2.1) 0.3–14.1
*p* values from HGLM and pairwise comparisons						
Outcome		Test for overall difference	T6 vs. T0	T12 vs. T6	T18 vs. T12	*p* for trend
MIDAS grade I		0.047	0.998	0.998	0.022	<0.001
MIDAS grade II		0.740	0.998	0.998	0.998	<0.001
MIDAS grade III		0.043	0.614	0.614	0.017	0.081
MIDAS grade IV		<0.001	<0.001	0.211	0.233	0.003

## Safety

During the onabotulinumtoxinA exposure, the unique reported treatment-related adverse event (AE) was neck pain. It occurred in only two patients from T0 to T12.

At baseline, six patients had received the endovenous treatment as a consequence of headache. During the study, the same patients were occasionally hospitalized in the emergency room due to poor treatment efficacy, but only one after T12.

## Discussion

Migraine is an episodic disorder, but in its natural course, its frequency, in a particular subset of patients, could progressively increase and evolve to a chronic form. The higher frequency of attacks can lead to a chronic intake of abortive medications as triptans and non-steroidal anti-inflammatory drugs (NSAIDs), both associated with a greater risk of developing cardio-cerebrovascular events and renal events [26, 27]. Thus, CM is a serious and debilitating neurological disorder with high risk of medication overuse given by the frequent partial response to treatment, both abortive and preventive. Therefore, the opportunity to provide new effective therapeutic options to patients represents a crucial step in CM treatment [28]. The PREEMPT clinical trials, the largest (1384 patients) studies in CM, showed that onabotulinumtoxinA is a safe, well-tolerated, and effective prophylactic therapy for CM patients [18–20].

The notable results led to the worldwide specific indication of onabotulinumtoxinA for the prevention of headache in CM patients [17–19, 29, 30].

Few studies in a real-life setting have been recently published and confirm the efficacy and safety of onabotulinumtoxinA [31–34]. Importantly among these, Negro et al. [18, 19] have proved in routinary practice that onabotulinumtoxinA can be safely used for long-term treatment of MOH comorbidity in CM. Furthermore, Guerzoni et al. [22] have described that therapy discontinuation leads to a general worsening of health-related quality of life.

Our retrospective study provides data from patients treated with onabotulinumtoxinA, administered over 18 months with six quarterly cycles, in a real-life setting. In our analysis, the efficacy of onabotulinumtoxinA is evaluated time by time, in addition to the comparison with baseline.

Our data show a significant decrease in the monthly headache days and hours, at each study evaluation, with respect to previous ones. They showed that beyond the first year, a statistically significant difference in the monthly days of headache comparing T18 vs. T12 is observed, so confirming that repeated injections over time might give much better results in support of observations by Negro et al. [20, 21].

There are still questions open in the onabotulinumtoxinA treatment for CM, such as the possible superior efficacy of symptomatic medication effects after repeated cycles.

Nevertheless, an increased sensitization in pain processing [35] has been described in patients with medication overuse; through the inhibition of peripheral sensitization [36], also onabotulinumtoxinA may influence central mechanisms responsible for facilitation in pain processing [37]. For the first time, our study evaluated the changes of the overall drug intake (in units of drug consumption) and the latency time of symptomatics during the onabotulinumtoxinA cycles, suggesting that repeated cycles of onabotulinumtoxinA decrease significantly the dosage of any of them and very importantly also the latency time period (*p* for trend <0.001). This represents an important preliminary finding since reduction in the use of pain medication and abuse might also constitute an essential factor reducing cardiovascular risk in patients affected with CM [26].

Further analyses are ongoing in order to evaluate the drug reduction by amount and therapeutic class and/or their association to assess potential similarities and/or differences in benefits.

The PREEMPT study efficacy analyses included the proportion of patients with a 50% or more decrease from baseline in the frequency of headache days and, separately, headache episodes [17, 19]. In our observation, we considered the mean reduction of monthly headache days and cumulative headache hours. Furthermore, similarly to our results, in the study by Silberstein et al. 2015 [30], a PREEMPT post hoc analysis showed that a meaningful proportion of patients with CM treated with onabotulinumtoxinA who did not respond to the first treatment cycle responded in the second and third cycles of treatment. Our findings confirmed a progressive and remarkable trend of “first-time response” upon repeat treatments. In fact, we observed a high conversion rate from patients defined as “non-responders” and “partial responders” to “responders” and “high responders” throughout the period under consideration.

We assessed how many patients showed a reduction of the symptoms between 50 and 75% and how many patients were high responders (reduction >75%), at each evaluation time. In our analysis, a significantly higher percentage of patients had a more than 75% decrease from baseline in the frequency of headache days and hours, improving over the time, which supports the evidence that the benefits of regularly repeated treatment are meaningful to the patients (﻿proportion of high-responder patients at T18 in headache days: 57.4%; T18 vs. T12 *p =* 0.009; proportion of high-responder patients at T18 in headache hours: 89.4%; T18 vs. T12 *p =* 0.051﻿).

The benefits of the therapy found additional evidences also in terms of improvement in the quality of life.

Patients were asked to complete a MIDAS questionnaire. The MIDAS grade is based on responses to five questions about disability associated with headache in the previous 3 months [25]. The MIDAS is used in clinical research and in clinical practice. The reliability and validity have been demonstrated in a series of studies [38, 39]. Our quality-of-life evaluation includes the MIDAS GRADE patient distribution at each study time. The data confirm the efficacy results described above. The proportion of patients in MIDAS grade I (which corresponds to little or no disability) increases over time, with a statistically significant difference between 18 months and 12 months. At the same time, the proportion of patients in grade III (moderate disability) decreases significantly. Conversely, already at the T6, we observed a significant decrease of patients in grade IV (severe disability). Once again, the result suggests that there is a consistent trend of improvement with repeated injection.

The safety results that emerged from our study confirm the data of the previous studies [17–19].

## Conclusion

Overall, our real-life results showed efficacy and safety of repeated cycles of onabotulinumtoxinA 155–195 U in patients affected with CM with or without overuse medication, thus confirming the efficacy data from previous randomized clinical trials for CM prophylaxis and pointing out that a prolonged treatment of onabotulinumtoxinA (18 months, six quarterly cycles) is able to sustain and further improve significantly the efficacy results and the quality of life achieved at 6 and 12 months.

AE, adverse event; CI, confidence interval; CM, chronic migraine; HGLM, hierarchical generalized linear model; ICHD-III (beta), International Classification of Headache Disorders, Third Edition-BETA Version; MOH, medication overuse headache; NSAIDs, non-steroidal anti-inflammatory drugs; PREEMPT, Phase III Research Evaluating Migraine Prophylaxis Therapy; q1–q3, lower-upper quartiles; SD, standard deviation
